# Assessing the Filariasis Causing Parasites in Adult Mosquitoes and the Vector Mosquito Larval Breeding in Selected Medical Officer of Health Areas in Gampaha District, Sri Lanka

**DOI:** 10.1155/2021/6643226

**Published:** 2021-04-10

**Authors:** S. A. S. Pilagolla, L. D. Amarasinghe

**Affiliations:** Department of Zoology and Environmental Management, Faculty of Science, University of Kelaniya, Dalugama, Kelaniya 11600, Sri Lanka

## Abstract

The present study was conducted to determine the prevalence of filariasis causing parasites in adult mosquitoes and vector mosquito larval breeding in four Medical Officer of Health (MOH) areas in Gampaha district, Sri Lanka. Adult female mosquitoes at their resting places were collected using a prokopack aspirator operated twice a day from 7.00 am to 8.00 am and 8.00 pm to 9 pm in predetermined dates. Microfilarial worms in dissected mosquitoes were morphologically identified. Nine species of mosquitoes, namely, *Culex quinquefasciatus, Cx. pipiens, Cx. fuscocephala, Cx. gelidus, Armigeres subalbatus, Mansonia uniformis, Ma. annulifera, Aedes aegypti*, and *Ae. Albopictus*, were captured. A total of 1194 mosquito larvae were collected that belonged into three genera, namely, *Culex* (62.73%), *Armigeres* (25.62%), and *Mansonia* (11.64%), from blocked drains, polluted drains, blocked canals, large polluted water bodies, stagnant water bodies, marsh lands, rice field mudflats, and concrete pits. Large polluted water bodies (Shannon-Wiener diversity index/*H*' = 1.5591) were the most diversed habitat type. In breeding water, average pH mainly lied in between 6 and 8 and average dissolved oxygen ranged from 3 to 7 mg/L. *Cx. quinquefasciatus* and *Armigeres subalbatus* adult female mosquitoes captured from Kelaniya MOH area were positive for microfilariae and were identified as *Wuchereria bancrofti* and *Dirofilaria repens*, respectively. This study concludes possible lymphatic filariasis situation is in extremely very low level persistent (0.06%) where transmission cannot be sustained and is restricted only to isolated pockets in the study area. The zoonotic strains of filariasis causing subcutaneous dirofilariasis in humans by *Dirofilaria repen*s is continuing to survive due to the presence of stray dogs that serve as reservoir hosts.

## 1. Introduction

Lymphatic filariasis (LF) is a mosquito-borne disease of humans and a major cause of disability worldwide [1, 2]. The most common clinical symptoms of LF are hydrocele, lymphedema, and adenolymphangitis [2, 3]. Elephantiasis is the advanced stage of lymphedema that results sociopsychological problems to patients and their families [4, 5]. Causative agents of LF are several species of nematode parasites of the order Spirurida and family Onchocercidae, namely, *Wuchereria bancrofti*, *Brugia malayi*, and *B. timori* [6]. According to the WHO, *W. bancrofti* is responsible for 90% of all human LF infections [7]. LF is transmitted by different types of mosquitoes, for example, by the *Culex* mosquito, widespread across urban and semiurban areas, *Anopheles*, mainly found in rural areas, and *Aedes*, mainly in endemic islands in the Pacific [7]. When infected mosquitoes bite people, mature parasite larvae are deposited on the skin from where they can enter the body [7].

In Sri Lanka, the LF has been endemic for hundreds of years in eight districts, namely, Colombo, Kalutara, Gampaha, Galle, Matara, Hambantota, Kurunegala. and Puttalam, bordering to western coast, the area well known as the “filariasis belt” [8]. In this country, LF is caused by *Wuchereria bancrofti* and *Brugia malayi*. More than 90 percent of cases of LF known as urban bancroftian filariasis is due to *W. bancrofti* while the remainder known as rural brugian filariasis is largely due to *B. malayi* [9, 10]. Mosquito vectors reported to cause brugian filariasis in Sri Lanka are the three species of *Mansonia*, namely, *Ma. annulifera*, *Ma. uniformis*, and *Ma. indiana* [11]. They are among the 159 mosquito species belonging to 19 genera reported in Sri Lanka [12]. In addition, *Dirofilaria repens* causing cutaneous dirofilariasis transmit by a range of mosquito species namely *Aedes aegypti*, *Armigeres subalbatus*, *Mansonia uniformis*, and *Ma. annulifera* [13]. Cutaneous dirofilariasis mostly affects children [13]. However, the sole vector responsible for the spread of urban bancroftian filariasis in Sri Lanka is *Culex quinquefasciatus* mosquito [14]. It breeds prolifically in polluted waters such in blocked drains and mud flats characterized by low dissolved oxygen (DO) and high biological oxygen demand (BOD) [14, 15]. According to [7], Sri Lanka at present is in eliminating of LF as a public health problem [7]. This was as a result of Mass Drug Administration (MDA) implemented by the Antifilariasis Campaign of Sri Lanka in order to reduce the microfilariae density in the blood of infected individuals to levels where the mosquito vectors are no longer capable of transmitting them to new human hosts and to reduce the microfilaria prevalence in the community to levels where transmission cannot be sustained despite presence of mosquito vectors [16–18]. However, WHO recommended the country to continue surveillance efforts and intervention to clear residual infections in foci with persistent infections [2]. Moreover, researchers have recently detected *W. bancrofti* and *Brugia* spp. in isolated cases in selected locations of the Gampaha district, Sri Lanka [9, 19]. Further, [19] reported that *Brugia* spp. also been detected in stained blood smears of stray dogs. According to the authors, this re-emergent strain of *Brugia* spp. was detected after a quiescent period of four decades and suggestive as a zoonotic origin [19]. Thus, there is a pressing need to determine the low level persistence of lymphatic filariasis parasites in potential mosquito species and to understand their breeding habitat requirements in a high risk area of the country.

## 2. Materials and Methods

### 2.1. Study Area

**Figure 1:**
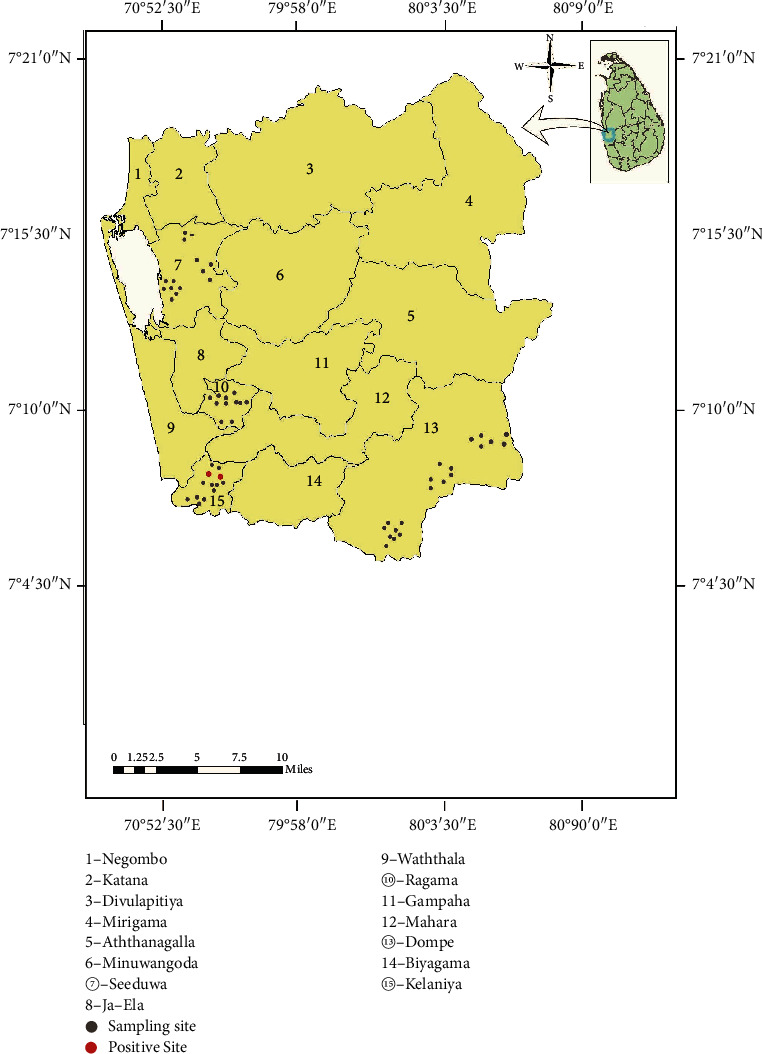
Map showing the selected MOH areas, sampling sites and positive sites for filariassis parasites in the Gampaha district.

Gampaha district is reported to have in the filariasis endemic area in Sri Lanka. It has a human population of about 2.4 million and spreads over an area of 1387 km^2^. Gampaha district receives an annual rainfall of about 2398 mm and an average annual temperature of 27.3°C (https://en.climate-data.org/asia/sri-lanka/western-province/gampaha-12453/). It is elevated at 25 amsl and boarded to western coast of Sri Lanka (Figure 1). The district consists of fifteen Medical Officer of Health (MOH) areas. Four MOH areas, namely, Kelaniya, Ragama, Seeduwa, and Dompe, were selected for this study (Figure 1).  Table 1 gives the brief description of the selected MOH areas.

### 2.2. Mosquito Sampling

Adult female mosquitoes were sampled at their natural resting places in three MOH areas using a battery-operated Prokopack aspirator (Model 1419; John W. Hock Co. Gainesville, FL, USA). Sampling was done twice a day, from 7.00 am to 8.00 am, and from, 8.00 pm to 9.00 pm, twice a week in predetermined dates from May to December 2019. Captured mosquitoes were immediately brought into the laboratory, sacrificed by cold shock and were sorted out by species [12, 20].

Potential mosquito larval breeding habitats were examined, and three dips each were taken into transparent wide mouth plastic bottles (300 mL) using a 250.0 mL larval scooper (width 11.5 cm, height 5.5 cm) fitted to a long metal handle [21] in two weekly intervals. The mean larval density was estimated. Water pH and dissolved oxygen (DO) were measured *in situ* using a multiparameter (HACH-HQ40d). Bottles containing mosquito larvae were brought into the laboratory and were covered using a small mesh sized nylon net (mesh size; 1 mm). Larvae were reared until the emergence of adult mosquitoes [22].

Adult female mosquitoes collected by Prokopack Aspirator and the emerged adults from larval rearing were identified up to a nearest possible taxonomic level, using standard mosquito identification keys [12, 20].

### 2.3. Adult Female Mosquito Dissection

Adult female mosquitoes collected by the Prokopack aspirator were placed in a 9 cm diameter Petri dish and their wings and thoracic legs were separated. Batches of ten to fifteen of them were placed on a small watch glass at once and a drop of buffered saline was added using a pasture pipette. Mosquito specimens were teased apart individually using two entomological pins under a stereo-microscope to reveal microfilariae in their midgut.

### 2.4. Staining Filarial Worms and Identification

Filarial worms on detection were carefully transferred onto a drop of saline and subsequently to a drop of water held on a microscopic glass slide. Specimen was heat fixed, and Giemsa staining was done [6]. Specimens were observed under a microscope *x* 400 magnification (OLYMPUS *x* C21; Jeff Liu Ningbo Huasheng Precision Technology International Trading Co., Zhejiang, China). Total length and length of cephalic space of microfilariae were measured using a microscopy digital USB camera (Optika 4083. B6) and OPTIKA version 2.12 image processing software. Identification was done based on [23, 14, 24]. Genera abbreviations were done based on [25].

### 2.5. Data Analysis

The statistical data analysis was performed using MINITAB 19 version. The mosquito species abundance data and physicochemical parameters in different larval habitats were analyzed using one-way ANOVA at 95% CI and at the significant level at *p* ≤ 0.05. Shannon-wiener diversity index was used to determine the diversity indices of the recorded mosquito species at different breeding habitats.

## 3. Results

### 3.1. Adult Mosquito Sampling

A total of 11702 adult mosquitoes were collected using prokopack aspirator and included *Culex quinquefasciatus*, *Cx. pipiens*, *Cx. gelidus Cx. fuscocephala*, *Mansonia uniformis*. *Ma. annulifera*, *Aedes aegypti*, *Ae. albopictus*, and *Armigerus subalbatus*, They were dominated by *Ar. subalbatus* (mean 1112 ± 555) followed by *Cx. quinquefasciatus* (mean = 816 ± 216) (Table 2). One-way ANOVA and Tukey's pair wise test revealed that adult mosquitoes of *Ar. subalbatus* were significantly higher than of *Cx. quinquefasciatus* (*F* = 3.47, DF = 8, *p* ≤ 0.05) (Table 2).

**Figure 2:**
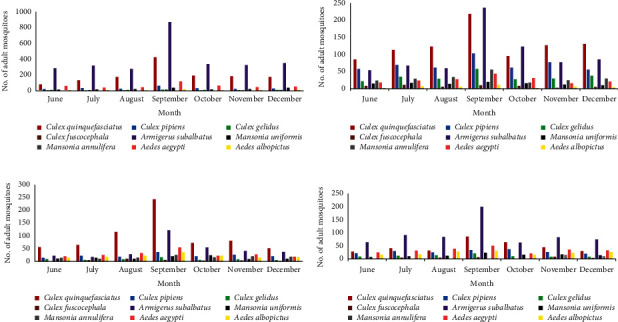
(a) Adult female mosquito composition in Kelaniya MOH area. (b) Adult female mosquito composition in Ragama MOH area. (c) Adult female mosquito composition in Seeduwa MOH area. (d) Adult female mosquito composition in Dompe MOH area.

The adult collections from Kelaniya and Dompe MOH areas were dominated by *Ar. subalbatus* (Mean = 393 ± 79.4 and 95 ± 18.3, respectively) (Figures 2(a) and 2(d)). One-way ANOVA and Tukey's pair wise test revealed that *Ar. subalbatus* monthly mean relative abundance was significantly higher than that of other mosquito species in the two MOH areas (*F* = 18.99, DF = 8, *p* ≤ 0.001 and *F* = 16.72, DF = 8, *p* ≤ 0.001, respectively). In Ragama and Seeduwa MOH areas, collected mosquitoes were dominated by *Cx. quinquefasciatus* (mean = 128 ± 16.5 and 97 ± 25.4, respectively) (Figures 2(b) and 2(c)).

### 3.2. Mosquito Larval Sampling and Breeding Habitats

Total 1194 mosquito larvae were captured during the study period of which 62.73% were *Culex*, 25.63% were *Armigeres*, and 11.64% were *Mansonia* (Table 3). Adult mosquitoes emerged from larval collection were identified as *Cx*. *quinquefasciatus, Cx. gelidus*, Cx. *pipiens*, *Cx. tritaeniorhynchus*, *Cx. fuscocephala*, *Ar. subalbatus*, and *Mansonia uniformis.* One-way ANOVA and Tukey's pair wise test revealed that *Cx*. *quinquefasciatus* and *Ar. subalbatus* monthly mean densities were significantly higher than those of other mosquito species (*F* = 6.02, d*f* = 6, *p* ≤ 0.001) (Table 3).

Larval habitats that came across during this study were blocked drains and canals, polluted drains, polluted and stagnant water bodies, marsh lands, rice field mudflats, and concrete tanks/pits. Mosquito density with an average level of pH and DO in their breeding habitats is given in Table 4. Large polluted water bodies (Shannon-Wiener diversity index/*H*' = 1.5591) and blocked drains (*H*' = 1.5381) were the highest divers habitat types and four species of mosquitoes, namely, *Cx. quinquefasciatus*, *Cx. pipiens*, *Cx. Fuscocephala*, and *Armigeres*, were reported in them (Table 4). All the *Culex* species larvae and *Ar. subalbatus* larvae were naturally distributed in low level of dissolved oxygen (3–5 mg/L). Only *Ma. uniformis* larvae which are totally depend on aquatic vegetation for their oxygen requirement were recorded in dissolved oxygen level higher than 5 mg/L. Larvae abundance, except *Cx. quinquefasciatus*, was reached to zero with the high level of DO content from 7 mg/L in natural breeding water. *Culex tritaeniorhynchus* was notably absent in many of the habitats reported to occupied by other culicine mosquitoes.

### 3.3. Mosquito Dissection for Microfilariae

*Ar. subpictus* and *Cx. quinquefasciatus* adult mosquitoes collected from Kelaniya MOH area (Figure 1) in September were positive for microfilaria worms in very low persistency at 0.0674% and 0.0613%, respectively, while other mosquito species were negative for any microfilariae (Table 5).

Microfilariae detected from *Ar. subalbatus* were identified as *Dirofilaria repens* by confirming to have unsheathed body, short cephalic space, and triangular shaped somatic cells while microfilariae that detected from *Cx. quinquefasciatus* were identified as *Wuchereria bancrofti* by having an ensheathed body, short cephalic space, tapered pointed tail, end without nuclei.

## 4. Discussion

A recent study in Sri Lanka reported the detection of brugian and bancroftian lymphatic filariasis parasites in humans after MDA [9], confirming the need to characterize the mosquito species involved in the low persistence of filariasis parasites in mosquitoes. In order to do that, we have addressed the following questions: (i) what species of mosquitoes are currently in abundance in high risk areas of Gampaha district of Sri Lanka? (ii) In which habitat type does these mosquitoes breed? (iii) Is there a temporal and spatial variation of mosquito abundance? and (iv) what are the mosquito species involved in LF transmission after MDA?

The only known vector for *Wuchereria bancrofti* in Sri Lanka is the night-biting mosquito, *Cx. quinquefasciatus* with their occurrence year-round [11]. *Culex quinquefasciatus* was recorded in relatively higher densities from their breeding habitats and adult sampling in Seeduwa MOH area during this study. Nevertheless, the most common mosquito species recorded in their breeding habitats and in adult sampling was *Ar. subalbatus*. *Culex fuscocephala* was recorded as one of the major species that breeds in marshland habitats. However, their adults captured by aspirator sampling did not report to carry filariasis parasites in this study. Although low densities of *Cx. gelidus*, *Cx. pipiens*, and *Cx. tritaeniorhynchus* larvae were recorded, the latter was not captured during adult collection.

*Culex quinquefasciatus* mosquito larvae did not sustain in marshland with vegetation and rice field mud flats, but they were flourished in blocked drains and polluted stagnant water bodies of those due to human activities. *Mansonia uniformis* thrived exceptionally in vegetation filled marshlands. Moreover, *Cx. tritaeniorhynchus* were only found in the rice field mud flats in the study area.

There is an empirical framework provided in this study for discerning the contribution of pH and dissolved oxygen level affecting to abundance and distribution of filarial and other mosquito larvae. It is reported that *Cx. quinquefasciatus* is predominant in organically polluted water bodies associated with human habitations such as blocked polluted drains [15]. According to the present study, 46% of *Cx. quinquefasciatus* and 44% of *Armigeres* were found in habitats with DO ranged between 3 and 5 mg/L. This approves the breeding grounds with appropriate physicochemical parameters, favour filariasis vector breeding. Mosquito larvae density in breeding habitats and the aspirator sampling of adult mosquitoes both had temporal variation over the sampling period from June to December. Adult mosquito collection reached to a peak during the month of September whereas the larvae collection was highest in the month of December showing a lag period of two months.

Entomological methods of filarial infection of vectors provide “real-time” estimates of filarial transmission [26]. This study evidently reports low level persistence of microfilaria in *Cx. quinquefasciatus* (0.0613 %) captured from Kelaniya MOH area in the month of September 2019. As reported by Pi-Bansa et al. [27], PCR assays may indicate microfilariae negative due to the masking of microfilarial DNA by mosquito DNA when the extraction is made using pooled mosquito samples. Parasite detection done using morphological characteristics of stained larvae in this study, though laborious, is accurate.

Results also indicate that filariasis is partially eliminated. and it is limited to isolated pockets in Sri Lanka at present. [28, 29], in their studies, have shown that even though microfilariae proliferate in mosquito body, parasite larvae cause lethal effect on mosquito host hence transmission from mosquito to human is interrupted.

Over the generations, mosquito species previously considered as nonvectors might be acting as vectors of filariasis parasites [30]. Hence, all the blood-fed mosquito species captured to the Prokopack aspirator collection were subjected to examine for microfilariae positivity. The Prokopack aspirator was the instrument utilized for adult mosquito surveillance for routing entomological surveys by the Regional Director of Health Services Office of Gampaha, Sri Lanka. This study reports the same vector mosquito involve in LF transmission after MDA, but there are zoonotic filarial worms proliferating to infect humans through *Armigeres* mosquito vectors.

## 5. Conclusions

Urban bancroftian and zoonotic filariasis are not in the zero level, and the latter is continuing due to existence of animal reservoirs. Varieties of breeding habitats with 7-8 pH and 3–5 mg/L DO levels are favoured by filariasis causing vector mosquitoes. This study confirms high abundance of *Ar. subalbatus* and *Cx. quinquefasciatus* in selected MOH areas in the Gampaha district, Sri Lanka. While former species involve in transmission of zoonotic filariasis parasites, the latter species involve in the low level persistence of bancroftian lymphatic filariasis parasites.

## Figures and Tables

**Table 1:** Descriptive information of four MOH areas.

	MOH area			
	Kelaniya	Ragama	Seeduwa	Dompe
Area covered (km^2^)	20	12	125	176
Population density (/km^2^)	6735	4223	3179	870
Vegetation/land cover	Marshes, paddy fields	Paddy fields	Marshes and mangroves.	Paddy fields and rubber plantations
Boundaries	Boarded to Kelani river	Surrounded by land cover	Boarded to Negombo lagoon	Surrounded by land cover

**Table 2:** Adult mosquito abundance in four MOH areas.

Mosquitoes species	MOH area				Mean ± SE
	Kelaniya	Ragama	Seeduwa	Dompe	
*Culex quinquefasciatus*	1357	900	680	326	816 ± 216^a^
*Culex pipiens*	234	490	156	194	268 ± 75.5^b^
*Culex gelidus*	65	240	58	82	111 ± 43.20^b^
*Culex fuscocephala*	80	51	30	28	47 ± 12.1^b^
*Armigeres subalbatus*	2756	708	320	665	1112 ± 555^b^
*Mansonia uniformis*	172	104	98	100	118 ± 17.90^b^
*Mansonia annulifera*	0	216	118	25	89 ± 49.10^b^
*Aedes aegypti*	423	182	196	237	259 ± 55.70^a^
*Aedes albopictus*	72	38	143	158	102 ± 28.60^b^

Same superscript in the column is not significantly different at *p* ≤ 0.05

**Table 3:** Monthly mean density of mosquito larval species.

Species	Month							Mean ± SE
	June	July	Aug	Sep	Oct	Nov	Dec	
*Culex quinquefasciatus*	25	32	51	28	40	30	70	39.43 ± 6.08^a^
*Culex gelidus*	3	11	15	20	12	9	40	15.71 ± 4.50^b^
*Culex pipiens*	12	7	22	9	19	13	25	15.29 ± 2.57^b^
*Culex tritaeniorhynchus*	5	16	14	12	9	8	19	11.86 ± 1.84^b^
*Culex fuscocephala*	20	25	31	12	33	17	21	22.71 ± 2.83^b^
*Armigeres subalbatus*	31	38	41	36	70	33	57	43.71 ± 5.44^a^
*Mansonia uniformis*	9	21	12	18	26	13	40	19.86 ± 4.01^b^

Same superscript in the column is not significantly different at *p* ≤ 0.05.

**Table 4:** Mean density of mosquito species larvae collected from different breeding habitats.

Habitat	Average pH	Average DO (mg/L)	Mosquito species						
			*Culex quinquefasciatus*	*Culex gelidus*	*Culex pipiens*	*Culex fuscocephala*	*Culex tritaeniorinchus*	*Mansonia uniformis*	*Armigerus subalbatus*
Blocked canals	6.96	6.88	0	0	17	51	0	0	50
Block drains	7.2	6.1	72	36	21	51	0	0	50
Polluted drains	6.76	5.9	113	0	34	0	0	0	37
Marsh land with vegetation	7.1	7.48	0	50	0	0	0	122	0
Large polluted water body with waste	7.31	5.8	32	0	20	34	0	17	41
Rice field mud flats	6.8	5.18	0	24	0	65	32	0	0
Stagnant water body	7.49	7.05	44	0	0	39	0	0	69
Concrete pits/tank	7.07	6.88	0	0	0	0	0	0	51
Other places	6.94	4.81	15	0	15	0	0	0	0

**Table 5:** Percentage of microfilaria (mf) positivity in adult mosquitoes.

Mosquitoes species	No of mosquitoes dissected	No. of positive mosquitoes for mf	Percentage of mf positivity
*Culex quinquefasciatus*	3263	2^*∗*^	0.0613
*Culex pipiens*	1074	0	0
*Culex gelidus*	445	0	0
*Culex fuscocephalus*	189	0	0
*Armigeres subalbatus*	4449	3^*∗*^	0.0674
*Mansonia uniformis*	474	0	0
*Mansonia annulifera*	359	0	0
*Aedes aegypti*	658	0	0
*Aedes albopictus*	411	0	0

^*∗*^Samples collected from Kelaniya in September.

## Data Availability

The datasets supporting the conclusions of this article are included in the article. Data will not be shared in any of the sources.
